# Granzyme B-Induced Neurotoxicity Is Mediated via Activation of PAR-1 Receptor and Kv1.3 Channel

**DOI:** 10.1371/journal.pone.0043950

**Published:** 2012-08-29

**Authors:** Tongguang Wang, Myoung-Hwa Lee, Elliot Choi, Carlos A. Pardo-Villamizar, Sung Bin Lee, In Hong Yang, Peter A. Calabresi, Avindra Nath

**Affiliations:** 1 Department of Neurology, Johns Hopkins University, Baltimore, Maryland, United States of America; 2 Section of Infections of the Nervous System, National Institute of Neurological Diseases and Stroke, National Institutes of Health, Bethesda, Maryland, United States of America; 3 Department of Biomedical Engineering, Johns Hopkins University, Baltimore, Maryland, United States of America; 4 Singapore Institute for Nanotechnology, National University of Singapore, Singapore, Singapore; University of Muenster, Germany

## Abstract

Increasing evidence supports a critical role of T cells in neurodegeneration associated with acute and subacute brain inflammatory disorders. Granzyme B (GrB), released by activated T cells, is a cytotoxic proteinase which may induce perforin-independent neurotoxicity. Here, we studied the mechanism of perforin-independent GrB toxicity by treating primary cultured human neuronal cells with recombinant GrB. GrBactivated the protease-activated receptor (PAR)-1 receptor on the neuronal cell surface leading to decreased intracellular cyclic AMP levels. This was followed by increased expression and translocation of the voltage gated potassium channel, Kv1.3 to the neuronal cell membrane. Similar expression of Kv1.3 was also seen in neurons of the cerebral cortex adjacent to active inflammatory lesions in patients with multiple sclerosis. Kv1.3 expression was followed by activation of Notch-1 resulting in neurotoxicity. Blocking PAR-1, Kv1.3 or Notch-1 activation using specific pharmacological inhibitors or siRNAs prevented GrB-induced neurotoxicity. Furthermore, clofazimine protected against GrB-induced neurotoxicity in rat hippocampus, *in vivo*. These observations indicate that GrB released from T cells induced neurotoxicity by interacting with the membrane bound Gi-coupled PAR-1 receptor and subsequently activated Kv1.3 and Notch-1. These pathways provide novel targets to treat T cell-mediated neuroinflammatory disorders. Kv1.3 is of particular interest since it is expressed on the cell surface, only under pathological circumstances, and early in the cascade of events making it an attractive therapeutic target.

## Introduction

T cell activation plays a critical role in brain inflammatory mechanisms and related neurodegeneration such as brain ischemic/reperfusion injury [1], multiple sclerosis (MS), acute disseminated encephalomyelitis, Rasmussen encephalitis [2], and HIV 1-associated immune reconstitution syndrome [3]. In MS, the extent of axonal damage is directly related to the number of infiltrating T cells in the white matter undergoing demyelination [4]. Activated T cells induce neurotoxicity through both cell contact-dependent [5] and –independent pathways such as release of granzyme B (GrB) [6]. GrB may cause perforin-independent cytotoxicity by using membrane receptors such as mannose-6-phosphate receptor and heparan sulfate containing receptor to facilitate its entry into the cells [7], [8] or by cleaving or interacting with the membrane-bound receptors such as glutamate receptor GluR3 [9] and Notch-1 [10] without entry into the cell. Notch-1 activation in neurons is related to neurotoxicity, especially neurite damage [11], [12]. We found that GrB-mediated neurotoxicity is pertussis toxin (PTX)-dependent [6], indicating a novel mechanism involving a G protein-coupled receptor (GPCR).

Protease-activated receptor-1 (PAR-1) is a serine protease activated GPCR found in specific neuronal populations and glial cells [13]. PAR-1 activation may exacerbate dopaminergic terminal damage [14] and neurite retraction in olfactory neurons [15]. PAR-1 activation-mediated neurite growth disturbance and caspase activation-dependent neuronal apoptosis have also been observed in motor neurons [16]. PAR-1 deficient mice were resistant to neuronal damage and neurologic deficits in a cerebral hypoxia/ischemia model [17], [18]. PAR-1 kinase-initiated tau phosphorylation may mediate neurite damage [19]. PAR-1 receptor is cleaved and activated by thrombin but it is still unknown whether GrB can activate PAR-1.

GrB inhibits neurogenesis by activating Kv1.3 channel on neural progenitor cells [20]. Kv1.3 is a *Shaker-*type delayed rectifier K+ channel found in immune cells including T-lymphocytes, dendritic cells, and microglia [21], [22] and its activation has been associated with excess immune responses and subsequent neurotoxicity [22], [23]. Kv1.3 antagonists can ameliorate experimental allergic encephalomyelitis [24], [25], an animal model of MS. However, Kv1.3 is also expressed in neurons [26] [27], [28] and its increased expression in cultured rat hippocampal neurons has been associated with neurotoxicty [29].

In the present study, we found that GrB may cleave PAR-1 in neurons, leading to its activation which is followed by increased expression of Kv1.3 channel and subsequent activation of Notch-1. Thus, we mapped a novel pathway of neurodegeneration and identified important therapeutic targets.

## Materials and Methods

### Human Fetal Neuron Cultures

All cell culture reagents were purchased from Invitrogen (Carlsbad, CA) if not otherwise specified. Human fetal neurons were cultured as previously described [30]. Briefly, human fetal brain specimens of 12–17 weeks gestation were obtained with permission of the Office of Human Subjects Research at the National Institutes of Health (NIH) and the Institutional Review Board at Johns Hopkins University. After removal of the meninges, brain tissues were triturated to single-cell suspension. Cells were then cultured in T75 flasks in opti-MEM with 5% (v/v) fetal bovine serum (FBS), 0.5% (v/v) N2 supplement and 1% (v/v) antibiotics. After reaching confluence, neurons were collected by carefully shaking the flasks and were reseeded at 1×10^5^/ml in 6-, 24- or 96-well poly-D-lysine- (Sigma, St. Louis, MO) coated plates for 1 week. At this stage, cultures contained 60–80% neurons and <1% microglia; the remaining cells were astrocytes as determined by immunostaining for beta-III-tubulin, CD68, and glial fibrillary acidic protein (GFAP), respectively.

### Cytotoxicity Assays

Cytotoxicity was evaluated using two methods. CellQuanti-Blue™ cell viability assay kit (BioAssay Systems, Hayward, CA) determined cell viability. Briefly, cells were cultured at 1×10^5^/ml in 90 µl serum-free opti-MEM medium in 96-well plates. The cells were treated with recombinant GrB (0.3–4 nM, EMD Chemicals, Gibbstown, NJ) with or without pretreatment with corresponding K+ channel blockers (rLq2, 100 nM; rBeKm-1, 10 nM; rTityustoxin-Kα, 100 nM and Dendrotoxin-K, 100 nM, Alomone Labs, Jerusalem, Israel) for 24 hr. Then, CellQuanti-blue solution (10 µl/well) was added for 30 min. Fluorescence intensity was then detected at the following wavelengths: Ex 530 nm, Em 590 nm. To assess neurite injury, cells cultured on poly-D-lysine-coated cover slips in 24-well plates were treated with GrB (4 nM) with/without PAR-1 inhibitor SCH 79797 (SCH, 50 nM; Tocris bioscience, Ellisville, MO), Kv1.3 inhibitor margatoxin (10 nM; Alomone Labs) or Notch-1 inhibitor L-685,458 (L, 10 µM; Sigma). After 24 hr of treatment, coverslips were collected for immunostaining for beta-III-tubulin. Beta-tubulin-positive cells were observed under a florescence microscope and images of nine pre-assigned fields were taken. The longest neurite length of each neuron was measured using open-lab software and the average neurite length from each group was compared.

### Immunocytochemistry

Cells were fixed in 4% (w/v) paraformaldehyde (Sigma) and then immunostained using monoclonal anti-beta-III tubulin (1∶1000; Promega), rabbit anti-GFAP (1∶1000; Sigma), rabbit anti-Kv1.3 (1∶100, Alomone), rabbit anti-active Notch-1(1∶100, Abcam, Cambridge, MA) and monoclonal anti-active caspase-3 (1∶100, Sigma) followed by corresponding secondary antibodies (anti-rabbit Alexa Flour 488, 1∶400 and anti-mouse Alexa Flour 597, 1∶400, Invitrogen) and DAPI nuclear staining. Images were acquired on a Zeiss LSM 510 META multiphoton confocal system (Carl Zeiss, Thornwood, NY) or a fluorescence microscope.

### Co-immunoprecipitation of GrB and PAR-1 Receptor

GrB antibody bead complexes were made by mixing 10 µl of monoclonal anti-GrB antibody (Chemicon, Themecula, CA) with 50 µl of protein G sepharose bead (GE Health, Chalfont St. Giles, UK) for 1 hr at room temperature. Human fetal neurons cultured in 6-well plates were treated with or without GrB (1 µg) for 10 min in 37°C and then at room temperature for 30 min. The cells were then lysed using RIPA buffer consisting of Tris 50 mM, NaCl 150 mM, sodium dodecyl sulfate (SDS) 0.1% (w/v), deoxycholate 0.5% (w/v) and NP-40 1% (v/v) (all components were purchased from Sigma). After centrifugation at 14,000 rpm. for 20 min, the supernatant was collected and incubated with GrB antibody bead column at 4°C overnight. The beads were washed three times with phosphate buffered saline, pH7.4 (PBS). After discarding the supernatants, SDS loading buffer was added to the beads and the mixtures were heated at 95°C for 10 min. Western-blot analysis was used to detect the PAR-1 receptor in the precipitates.

### Western-blot Analysis

Cells were lysed with RIPA buffer for Western-blot analysis of PAR-1, Kv1.3, and activated Notch-1. Protein concentrations were determined with the BCA Assay kit (Pierce, Rockford, IL, USA) following the manufacturer’s instructions. Equal amounts of protein (20 µg per lane) were separated by 10% (w/v) Tris-glycine polyacrylamide gels and transferred to polyvinylidene difluoride (PVDF) membranes. Membranes were blocked with 10% (w/v) non-fat milk and incubated with monoclonal anti-PAR-1 antibody (1∶500, Abcam), rabbit anti-Kv1.3 antibody (1∶100), rabbit anti-active Notch-1(1∶500, Abcam), rabbit anti-Notch-1 antibody (1∶200, Santa Cruz, CA) and monoclonal mouse-anti-β actin (1∶5000, Sigma) overnight at 4°C. After washing, membranes were incubated with peroxidase-linked anti-rabbit or anti-mouse IgG (1∶5000; GE healthcare) for 1 hr at room temperature. Amersham ECL™ Western Blotting Detection Reagents (GE Healthcare) were used for detection. Optical density of the bands was measured using NIH ImageJ software.

### Cyclic AMP Detection

Primary cultured human neuronal cells in 24-well plates were treated with GrB (4 nM) for 15 min with or without PAR-1 inhibitor SCH pretreatment. The cells in each well were then lysed in 500 µl lysis reagent contained in the kit and intracellular cAMP levels were measured using Amersham cAMP Biotrak enzyme immunoassay system (GE Healthcare) according to manufacturer’s instructions.

### Intracellular K^+^ Concentration Detection

Intracellular K^+^ concentration was measured as published previously [31], [32] with modifications. Briefly, 10 mM of potassium-sensitive K^+^-binding benzofuran isophtalate (PBFI) stock was made by dissolving 50 ug PBFI AM (Invitrogen) in 20% (w/v) Pluronic F127 solution in DMSO (Invitrogen). Human fetal neurons in 96-well plates were washed three times with K free solution (containing 159 mM NaCl, 3.6 mM NaHCO_3_,1 mM MgCl_2_, 5 mM HEPES, 2.3 mM CaCl_2_, 10 mM glucose; pH 7.4) before incubation with 5 µM PBFI AM for 1 hour. After washing with the K free solution, the cells were treated with GrB (10 nM) for 2 hours with/without 30 min of MgTX (10 nM) pretreatment. Intracellular K^+^ concentration was measured by detecting the fluorescence intensity at the following wavelengths: Ex 340 nm, Em 500 nm.

### Inhibition of Kv1.3 and PAR-1 Expression with siRNA

Human fetal neurons in 24-well plates were used to transfect siRNA. Kv1.3-specific siRNA, PAR-1-specific siRNA, and a nonspecific negative control siRNA were purchased from Dharmacon (Chicago, IL). Briefly, 1 µl of transfectamine 2000 (Invitrogen) was added to Opti-MEM I reduced serum medium to a final volume of 50 µl. 0.2 µl of 50 µM siRNA was added to Opti-MEM I to a final volume of 50 µl and incubated for 5 min. The siRNA and transfectamine solutions were combined and mixed by gentle pipetting and incubated at room temperature for 20 min. Cells were washed with Opti-MEM I, and 400 µl of fresh Opti-MEM I were added to each well. The transfection agent/siRNA complex was added dropwise onto the cells and incubated for 48 hr at 37°C after which the cells were ready for further GrB treatment.

### CBF1 Transactivation Assay

8X wild type and control mutant CBF1-luciferase reporter constructs were provided by Dr. Diane Hayward (Johns Hopkins University). HEK293 cells cultured in 24-well plates in serum and antibiotic free Opti-MEM were transfected with 0.8 µg of either 8XwtCBF1Luc or 8XmtCBF1Luc plasmids using lipofectamine 2000. The media was replaced with fresh DMEM with 5% (v/v) FBS after 48 hr and the cells were treated with GrB (4 nM) with/without PTX (100 ng/ml) or MgTX (100 nM). Luciferase activity was detected after 48 hr using a luciferase assay kit from Stratagene (La Jolla, CA) according to the manual.

### Animals and Procedures

Eight-week-old female Sprague-Dawley rats purchased from Charles River Laboratories (Raleigh, NC) were randomly divided into three groups for stereotaxic injection into the hippocampal dentate gyrus (DG). Group I (n = 5) received GrB injection in the DG. Clofazimine (Sigma, 50 µg/kg/day) was injected intraperitoneal (clofazimine was first dissolved in DMSO as 10 mg/ml solution and then diluted in PBS as 10 µg/ml working solution) for 10 days (three days before and seven days after the GrB injection). Group II (n = 4) received GrB injection in the DG and vehicle by intraperitoneal injection. Group III (n = 4) received control solution both by DG injection in the DG and intraperitoneally. In these studies, we focused on the quantification of the doublecortin (DCX)-positive cells in the DG. DCX is expressed only in newly generated immature neurons [33] which might have been the majority of cells in our primary culture system. Also, the limited numbers of DCX-positive neurons in the DG made it easy for us to study neurite length and cell numbers, which are technically difficult to delineate in normal dense DG neuronal populations.

### Immunohistochemistry

Brain tissues obtained at autopsy from four patients with a definite diagnosis of MS were obtained from the Brain Repository Center, Division of Neuroimmunology and Neuroinfectious Diseases at The Johns Hopkins University Department of Neurology. Four healthy control brain samples were obtained from the Cooperative Human Tissue Network, Charlottesville, VA. Paraffin sections were deparaffinized, heated in a pressure cooker in 10 mM citric acid (pH 6.0) for 1 min, treated with 4% (v/v) normal goat serum/0.4% (v/v) Triton X-100/Tris-buffered saline (TBS) for 1 hr and incubated overnight with anti-Kv1.3 (1∶100, Alomone Laboratories) or anti-CD4 antibodies. Sections were rinsed with TBS and incubated with biotin-conjugated secondary antibody followed by Vectastain Elite ABC and diaminobenzidine (DAB) (both from Victor Labs, Burlingame, CA). Nuclei were counter-stained with Harris’s hematoxylin (Sigma). Controls were prepared by immunostaining without the primary antibody, by using control isotype IgG.

### Preparation of Compartmentalized Chambers

45 g of polydimethylsiloxane (PDMS) and 5 g of formaldehyde curing agent were mixed and poured over micro fabricated wafer, and baked overnight. PDMS replicas were cut into 5×2 chambers, with holes punched out near the micro channels. Chambers were plasma bonded onto glass plates. Finished chambers were autoclaved, and each well was washed three times with 100% ethanol and three times with sterile water. The chambers were then coated with PDL.

### Mouse Neuronal Cultures

Mouse cortical neurons were purchased from brainbitsllc.com. Upon arrival, cells were plated onto somal wells (one side of the chamber only) and incubated for five days in neurobasal media containing B27 supplements, Glutamax and antibiotics (all from Invitrogen).

### T Cell Activation and Treatment

T cells were cultured and activated as previously reported [6]. T cell supernatants (1∶5 dilution in neural basal media) were used to treat the neuronal cultures either on the somal side or on the axonal side. For control, cells were treated with T-cell media diluted in neurobasal media. Phase-contrast images of axons were taken before and 72 hours after the treatment.

### Statistical Analysis

Statistical analysis was performed using PRISM version 3.0. Results were analyzed using Student’s t test or one-way ANOVA for multiple comparisons. Two-tailed p-values <0.05 were considered significant.

## Results

### GrB Induced Neurotoxicity by Reducing Cell Numbers and Neurite Lengths

**Figure 1:**
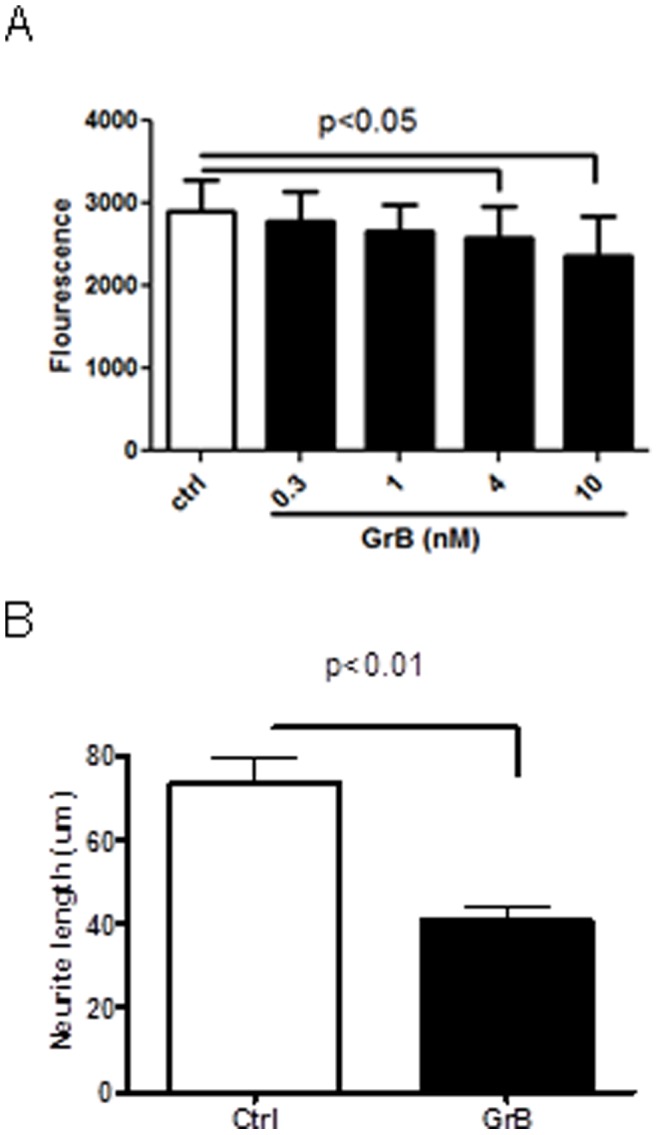
GrB induces neurotoxicity. (A) Human fetal neurons cultured in 96-well plates were treated with GrB (0.3–10 nM) for 24 hr and cell viability was measured using Cellquanti-blue assay. Results represent average ± SEM from four independent fetal cultures. (B) Human fetal neurons on coverslips in 24-well plates were treated with GrB (4 nM) for 24 hr and neurons were immunostained for beta-III-tubulin. Average neurite lengths were measured as described in the methods section. Results represent average ± SEM from three independent experiments.

To determine the effect of GrB on neurons, primary cultures of human fetal neurons were treated with recombinant GrB (0.3–10 nM) for 24 hr. Cell viability was measured by a fluorescence-based Cellquanti-blue assay. GrB caused neurotoxicity in a concentration-dependent manner, with up to 20% reduction in viable cells at 10 nM of GrB (Figure 1A). We observed that using a chamber culturing system which separated neuronal cell bodies and axons, the supernatants from activated T cells caused more significant damage to axons when they were added to the cell body side than when they were applied to the axon side (Figure S1). Thus, we studied the effect of GrB on axonal toxicity by adding GrB to the culture media.. Neurons were immunostained for beta-III-tubulin. GrB treatment led to about 40% reduction in average neurite length (Figure 1B). Thus, GrB caused neuronal death in a subpopulation of neurons and more recognizable damage to neurites.

### GrB Activated PAR-1 by Binding and Cleavage of the Receptor

**Figure 2:**
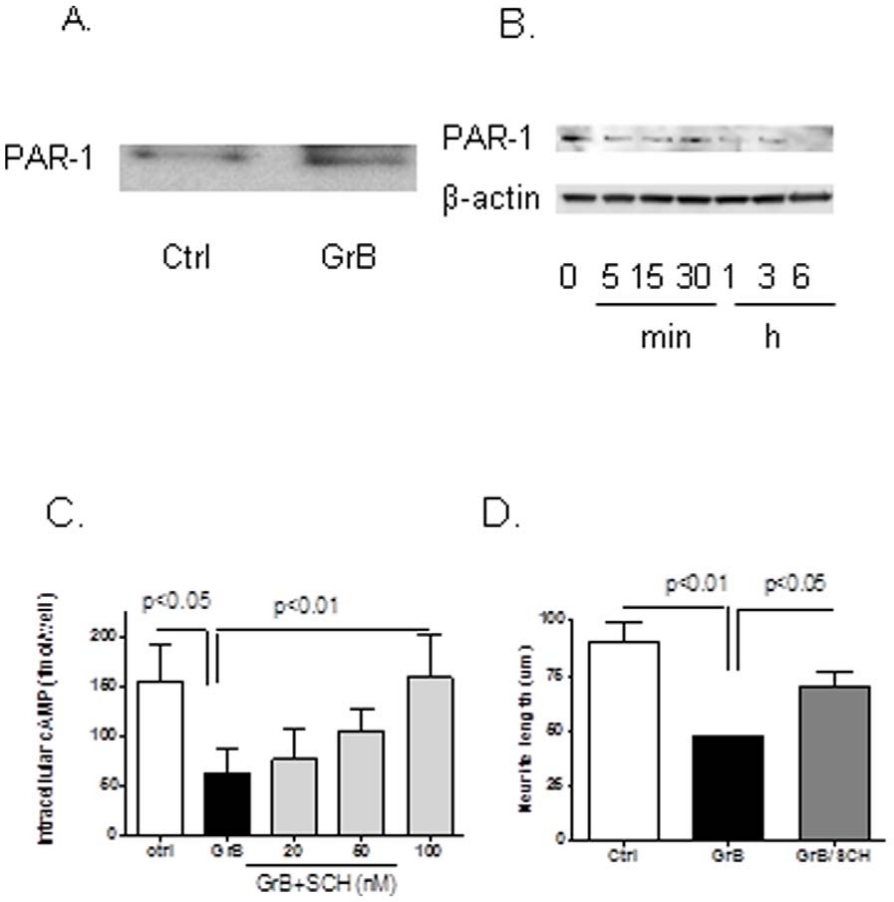
GrB activates PAR-1 receptor. (A) Human neuronal cells were treated with or without GrB for 30 min and then lysed with RIPA buffer. Cell lysates were incubated with GrB protein-bound protein G beads overnight at 4°C. After washing, immunoprecipitates were resolved by SDS-PAGE and subjected to Western blot analysis using a PAR-1 specific antibody. A representative blot of three independent experiments is shown. (C) Human neurons were treated with GrB cell lysates were collected for PAR-1 detection using Western-blot analysis. A representative blot of three independent experiments is shown. GrB treatment diminished PAR-1 protein as early as 5 min following exposure, indicating that GrB may cleave the PAR-1 receptor, a classical way for PAR-1 activation. (D) Intracellular cAMP level was detected 15 min after GrB treatment in the presence and absence of SCH, a PAR-1 inhibitor. Results represent average ± SEM from six independent experiments. (E) Effect of PAR-1 activation on GrB-induced neurotoxicity was studied by quantifying neurite length. Human fetal neurons on coverslips in 24-well plates were treated with GrB (4 nM) with or without SCH (50 nM) pretreatment. After 24 hr, neurons were immunostained for beta-III-tubulin. Average neurite lengths were measured as described in the Methods section. Results represent average ± SEM from three independent experiments.

To determine whether GrB interacted with PAR-1 receptor, co-immunoprecipitation was performed. Neuronal extracts were incubated with GrB immune complexes bound to a protein G column. Protein complexes were analyzed by Western-blot analysis using antisera to PAR-1. PAR-1 co-precipitated with GrB, indicating that GrB binds to PAR-1 receptors on the cell membrane (Figure 2A).

The effect of GrB on PAR-1 was also studied after 5 min to 6 hr of GrB treatment using Western-blot analysis. GrB treatment decreased PAR-1 protein significantly, in a time-dependent manner, starting as early as 5 min and reaching a peak around 6 hr. The immediate decrease of PAR-1 indicates a degradation of protein instead of inhibition of expression. It is in agreement with the pattern of PAR-1 activation by cleavage. (Figure 2B). The involvement of PAR-1 activation in T cells mediated neurotoxicity was also confirmed as we observed that treatment with supernatants of activated T cells also resulted in decreased PAR-1 on human neurons (Figure S2). We previously showed that GrB caused neurotoxicity by decreasing intracellular cAMP levels, an effect that can be measured within a few minutes. Hence, we determined if PAR-1 specific blocker SCH could block the effects of GrB on cAMP levels. Primary cultures of human neurons were pretreated with SCH for 30 min prior to GrB treatment. After 15 min of GrB treatment, cell lysates were collected for intracellular cAMP determination. We found that while GrB treatment decreased cAMP level, pretreatment with SCH significantly dose-dependently attenuated the effect with complete blockage at the highest dosage, confirming the role of GrB-PAR-1 interactions in mediating these effects (Figure 2C). SCH also attenuated the effects of GrB on neurite retraction. These results indicate that GrB may directly bind and cleave PAR-1, activating the receptor to cause neurotoxicity.

### Kv1.3 Channel Activation Mediated GrB-induced Neurotoxicity

**Figure 3:**
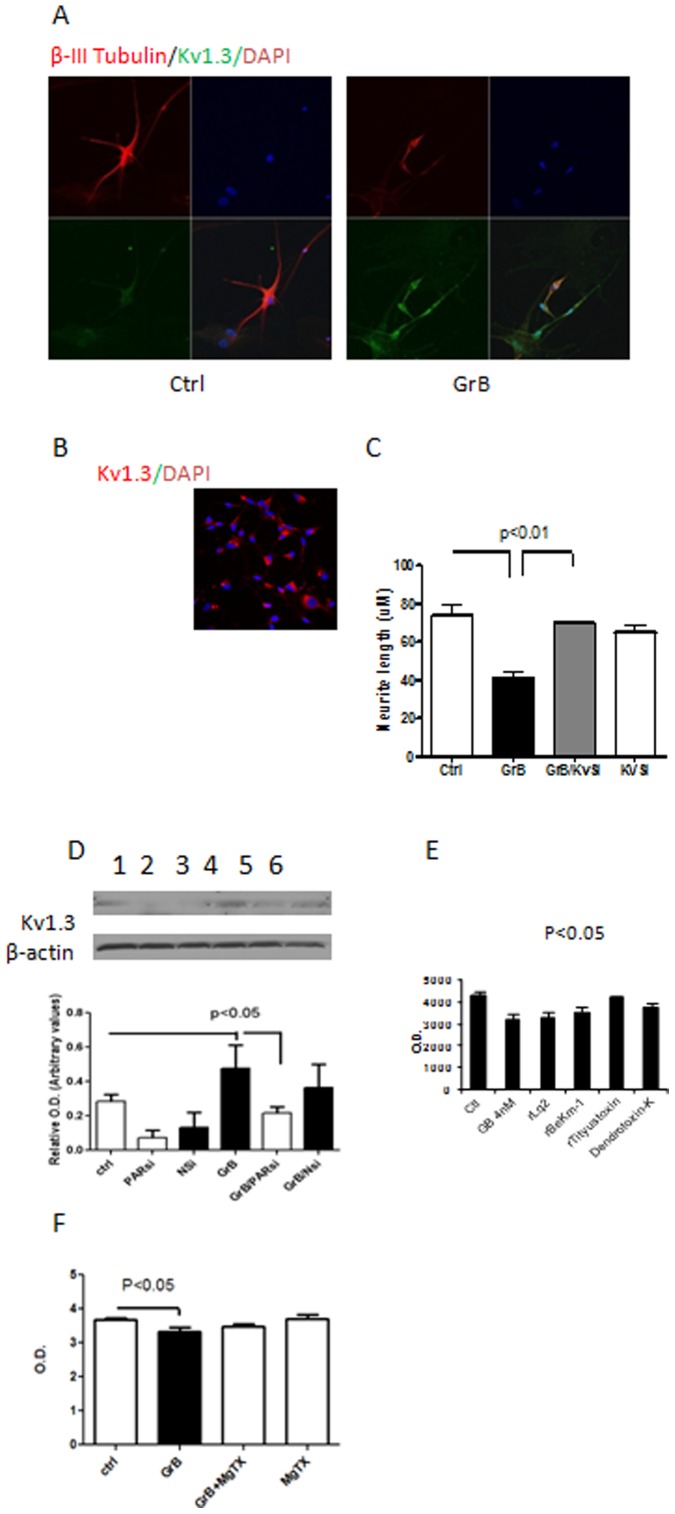
GrB activates Kv1.3 channel in neurons. (A) Human fetal neurons were treated with GrB (4 nM) for 24 hr. Cells were then fixed and immunostained for Kv1.3 and beta-III tubulin and analyzed by confocal microscopy. Representative photomicrographs from three independent experiments with similar results are shown. (B) Human fetal neurons were pretreated with cycloheximide (CHX, 100 µg/ml) or actinomycin D (Act D, 10 µM) for 30 min prior to GrB (4 nM) treatment. 24 hr later, cells were fixed and immunostained for Kv1.3 and analyzed by confocal microscopy. Representative photomicrographs from three independent experiments with similar results are shown. (C) Primary human neuronal cultures were first transfected with siRNA specific to Kv1.3 (KvSi). After 48 hr, GrB (4 nM) was used to treat the cells. Cells were fixed after 24 hr and immunostained for beta-III-tubulin. Neurite lengths were measured as detailed in Methods. Results represent average ± SEM from three independent experiments. (D) Human neuronal cells were transfected with PAR-1 specific siRNA (PARsi) or a nonspecific control siRNA (Nsi) 48 hr prior to GrB treatment and Western-blot analysis was used to detect Kv1.3 expression after 24 hr of GrB treatment. Representative blot is shown (Lane 1: control; lane 2: PARsi; lane 3: Nsi; lane 4: GrB; lane 5: GrB/PARsi: Lane 6: GrB/Nsi) and results are presented as average ± SEM from three independent experiments. (E) Primary human neuronal cultures were pretreated with corresponding inhibitors 30 min prior to GrB treatment (4 nM). Cell viability was determined using Cytoquantiblue assay 24 hr later. Results represent mean ± SEM. (F) Cells were incubated with a K free solution containing 5 uM PBFI AM for 2 hours. After washing, the cells were treated with GrB (10 nM) with/without MgTX (10 nM) pretreatment. Intracellular K+ concentration was determined by measuring the florescence at Ex 340 nM and Em 500 nM. Data represents mean ± SEM from five replicates.

We next studied whether Kv1.3 channel played a role in GrB-induced neurotoxicity. By immunostaining, we found that GrB treatment increased Kv1.3 expression in cells with shorter neurites; some of the cells exhibited an apoptotic appearance, such as fragmented nuclei (Figure 3A). The increased Kv1.3 expression was also observed in neurons treated with supernatants from activated T cells (Figure S3). Furthermore, pretreatment with cycloheximide (CHX, 100 µg/ml) an inhibitor of protein synthesis attenuated GrB-induced Kv1.3 expression suggesting that the increased expression of Kv1.3 following GrB treatment was due to new protein synthesis and not due to translocation of the protein from cytoplasm to the cell membrane. Pretreatment with actinomycin D (Act D, 10 µM) had a moderate result, suggesting that the effect of GrB on Kv1.3 may be regulated at the level of transcription (Figure 3B). These observations were further confirmed by pretreating the neurons with Kv1.3-specific siRNA, leading to a significant attenuation of GrB-induced neurite shortening (Figure 3C). Furthermore, when using Western-blot analysis to monitor Kv1.3 expression, PAR-1 -specific siRNA transfection was found to completely block GrB-induced Kv1.3 increase and even decreased its basal level expression in control cells (Figure 3D), indicating that (a) PAR-1 mediates Kv1.3 activation; and (b) Kv1.3 activation plays an important role in mediating GrB-caused neurotoxicity. To determine whether the protective effect of Kv1.3 inhibition is specific for this channel, we also pretreated the human fetal neurons with inhibitors of other K channels such as rLq2 (100 nM) which specifically blocks inward rectifier Kiv1, rBeKm-1 (10 nM) which specifically blocks ERGI K+ channels, rTityustoxin-Kα (100 nM) which is a non-specific voltage-gated K+ channels blocker, and Dendrotoxin-K (100 nM) which specifically blocks the voltage-gated Kv1.1 channel. This was followed by GrB (4 nM) treatment; cytotoxicity was monitored using cytoquanti-blue assay 24 hr later. Only rTityustoxin-Kα significantly protected GrB-induced cytotoxicity (Figure 3E), indicating GrB-caused cytotoxicity is Kv1.3-specific. We also measured the effect of GrB on intracellular K^+^ concentration using the PBFI assay. The PBFI assay was calibrated first with known extracellular K^+^ concentrations of 0 to 160 mM in 40 mM increments by substituting Na^+^ for K^+^ in a K free solution (supplemental Figure S4). As shown in Figure 3F, GrB treatment caused a significant decrease in intracellular K^+^ concentration compared to the control cells, but not in cells pretreated with MgTX, indicating that the depletion of intracellular K^+^ may also play a role in GrB-induced neurotoxicity.

### Notch-1 Activation is Required for GrB–induced Neurotoxicity

**Figure 4:**
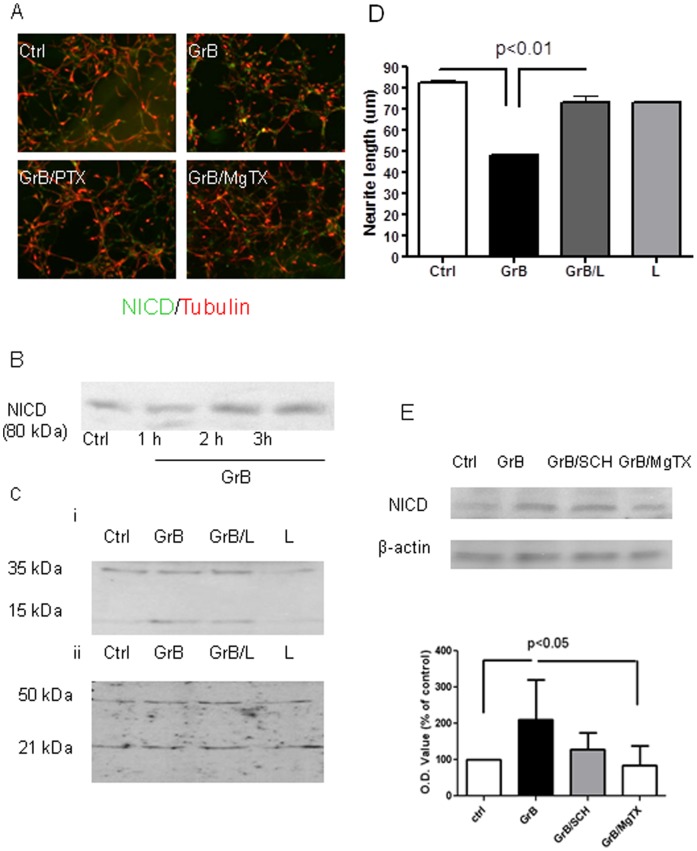
GrB activates Notch-1 receptor. (A) Human fetal neuronal cultures were treated with GrB (4 nM) with or without PTX (100 ng/ml) and MgTX (10 nM) for 18 hr. Cells were then fixed and immunostained for NICD and beta-III tubulin. Representative photomicrographs from three independent experiments with similar results are shown. GrB treatment for 18 hr increased intracellular Notch-1 activation in neurons, while pretreatment with PTX (100 ng/ml) or MgTX (10 nM) both significantly attenuated the effect. (B) Western-blot analysis showed that GrB increased Notch-1 activation in a time-dependent manner and NICD production reached peak within 3 hr. Representative blots from three independent experiments with similar results were shown. (C) Primary cultured human neurons were treated with GrB (4 nM) for 24 hr with the supernantants (sups) and cell lysates collected separately. Cell lysates (i) and concentrated sups (ii) were then used for Western-blot analysis. By using NICD antibody, an extra15 kDa band was found in the GrB-treated cell lysates which was attenuated by pretreatment with Notch-1 inhibitor “L” (10 µM). No significant changes were noted in the bands from the sups when using an antibody specific to C-terminal extracellular Notch-1 fragments. Representative blots from three independent experiments with similar results are shown. (D) Human fetal neuronal cultures were treated with GrB (4 nM) with or without “L” (10 µM) pretreatment. After 24 hr, neurons were immunostained for beta-III-tubulin and average neurite lengths were measured. Results represent average ± SEM from three independent experiments. (E) Human fetal neuronal cultures were pretreated with SCH (50 nM) and MgTX (10 nM) 30 min prior to GrB (4 nM) incubation. Cell lysates were collected 3 hr later for Western blot analysis using an antibody specific to NICD. Results represent average ± SEM from five independent experiments. A representative blot is shown.

**Figure 5:**
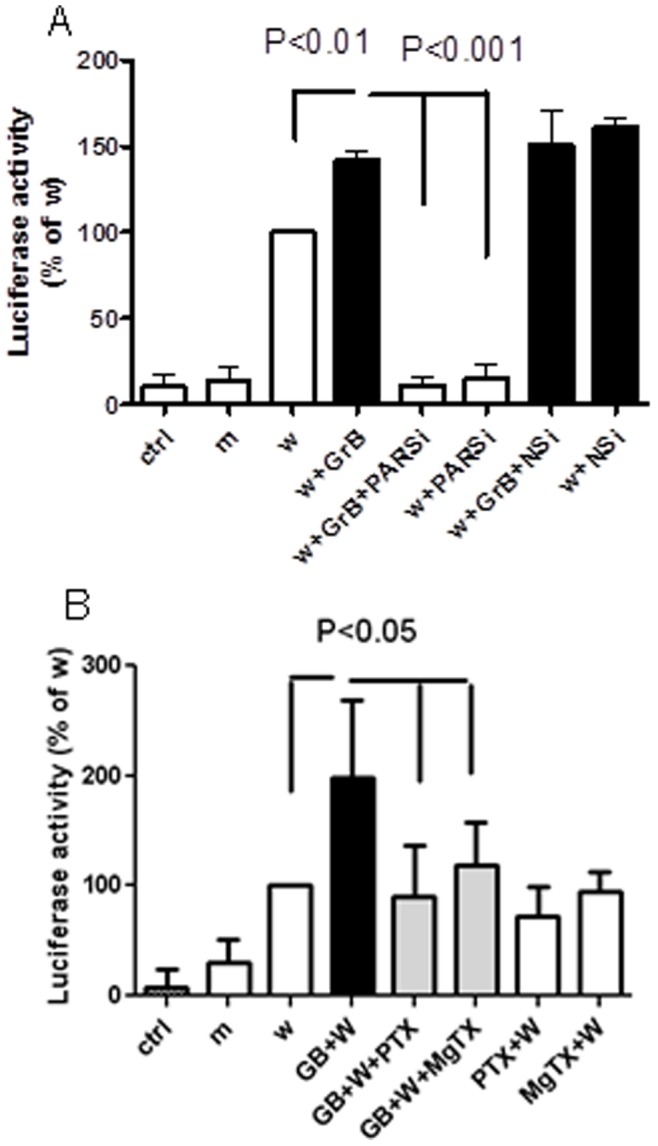
Notch-1 pathway activation by GrB is mediated by PAR-1 and Kv1.3. Confluent HEK293 cells were transfected with wild 8XCBF-1 luciferase construct (w) or mutant control construct (m). (A) Cells were co-transfected with PAR-1 specific siRNA (PARSi) or negative siRNA (NSi) to determine the effect of PAR-1 on Notch-1 activation. After 48 hr, cells were treated with GrB (4 nM). Cell lysates were collected 48 hr later and luciferase activity was quantified. Results are average ± SEM from three independent experiments. (B) In another set of experiments, 48 hr after transfection, cells were treated with GrB (4 nM) with or without 30 min pretreatment with PTX (100 ng/ml) or MgTX (100 nM). Luciferase activity was quantified after an additional 48 hr. Results represent average ± SEM from six independent experiments.

Notch-1 is known to mediate neurotoxicity, especially neurite growth. Also it has been recently reported that GrB may directly cleave Notch-1 (12). Therefore, we investigated if Notch-1 was involved in GrB-induced neurotoxicity. We initially treated the human neuronal cultures with GrB and then immunostained the cells with antibodies specific to the Notch-1 intracellular active fragment or the Notch intracellular domain (NICD) and beta-III-tubulin. GrB treatment resulted in increased NICD-positive neurons which could be attenuated by pretreating with pertussis toxin (PTX), a blocker of Gi protein-coupled receptors and MgTX, a Kv1.3-specific blocker (Figure 4A). To specify whether GrB activated Notch-1 by direct cleavage or not, we collected supernatants and cell lysates for Western-blot analysis at different time points after GrB treatment. GrB increased Notch-1 activation in a time-dependent manner and NICD production peaked within 3 hr (Figure 4B). The increased NICD production was also observed in activated T cell supernatants treated neurons, compared to control T cell supernatants (Figure S3). By using an antibody to NICD, an additional 15 kDa band was found in the GrB-treated cell lysates at 24 hr, which could be attenuated by pretreatment with a Notch-1 inhibitor, indicating it may be a cleaved NICD fragment (Figure 4Ci). However, no significant change in the bands was observed in the supernatants when using an antibody specific to the C-terminal extracellular Notch-1 fragment, indicating that there was no direct cleavage of Notch-1 extracellularly (Figure 4Cii). To determine whether GrB-induced NICD fragments were active, we used a Notch-1 inhibitor to modulate GrB-mediated neurotoxicity. Notch-1 inhibition significantly attenuated GrB-induced neurite shortening, indicating Notch-1 activation was involved in GrB-mediated neurite damage (Figure 4D). To further delineate the mechanism of Notch-1 activation in GrB-treated cells, we transfected HEK293 cells with a CBF-1 luciferase construct. When the Notch pathway is activated, the resulting CBF-1 transcription leads to increased luciferase production. GrB treatment in the transfected cells further increased luciferase activity, while PAR-1-specific siRNA, PTX and MgTX pretreatment significantly attenuated GrB-induced luciferase activity (Figure 5). These observations suggest that functional Notch-1 activation was mainly a secondary event instead of direct GrB cleavage, and likely an event downstream of Kv1.3 activation.

### Kv1.3 Activation is Specific to GrB-induced Neurotoxicity

**Figure 6:**
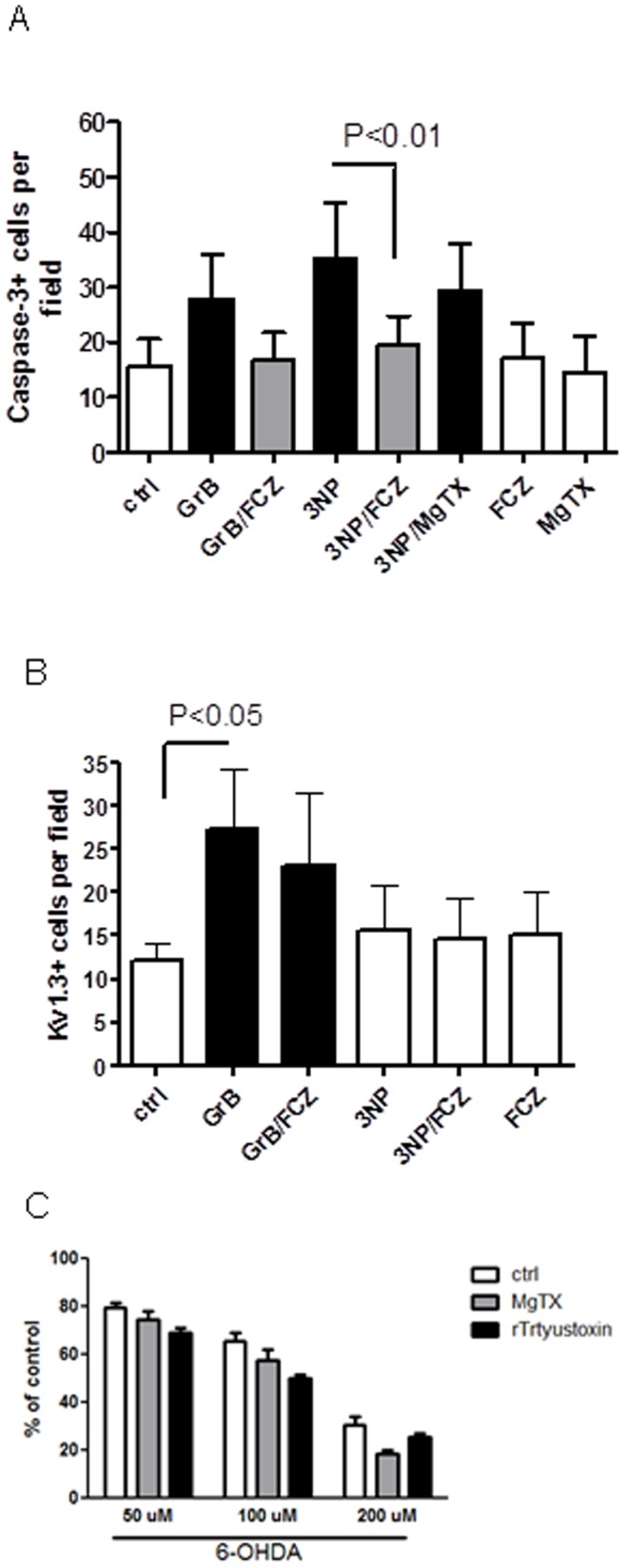
Kv1.3 activation is specific to GrB-mediated neurotoxicity. A. Human neuronal cultures were treated with GrB (4 nM) and mitochondrial inhibitor 3NP (3 mM) with/without 30 min of pretreatment of MgTX (10 nM) or fluconazole (FCZ, 10 µM). After 24 hr, cells were collected and fixed. Duplicate coverslips from each treatment were immunostained for active caspase-3 and Kv1.3. Positive cells in each of nine predesigned fields were counted. Average of positive cells in each of the fields was calculated. Results represent average ± SEM from three independent experiments. B. Human neuronal cultures were treated with 6-OH-dopamine (50–200 µM) with/without 30 min of pretreatment of MgTX (10 nM) or rTrtyustoxin (10 nM). After 24 hr, cell viability was measured using Cellquanti-blue assay.

To determine whether Kv1.3 activation is specific to GrB-induced neurotoxicity, we treated human primary cultured neurons with GrB and mitochondrial inhibitor 3-nitropropionic acid (3NP) with/without pretreatment of MgTX or fluconazole, a known compound that can protect neurons against 3NP-induced neurotoxcity in our culture system. After 24 hr, the cells were immunostained for active caspase-3 and Kv1.3. We found that both GrB and 3NP treatment increased the number of active caspase-3 positive cells and both effects were attenuated by fluconazole pretreatment (Figure 6A). But only GrB increased the number of Kv1.3 positive cells. While MgTX pretreatment decreased GrB-caused caspase-3 activation it had no effect on 3NP-induced caspase-3 activation (Figure 6B). We also tested the effect of Kv1.3 inhibition on 6-OH-dopamine caused neurotoxicity (Figure 6C). 6-OH-dopamine induced a concentration-dependent neurotoxicity, neither MgTX or rTrtyustoxin pretreatment attenuated the toxicity. These observations indicate that Kv1.3 activation shows some specificity for GrB-induced neurotoxicity and occurs before mitochondrial inhibition.

**Figure 7:**
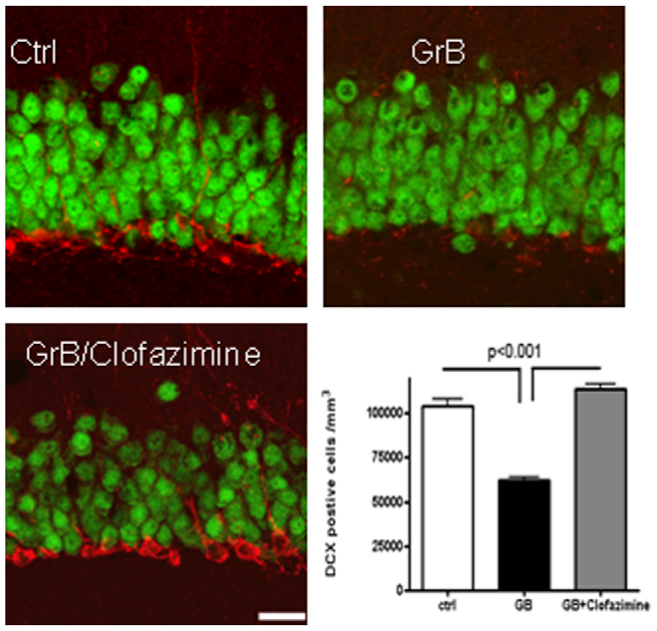
Clofazimine protected against GrB toxicity in hippocampal neurons *in vivo*. Eight-week-old female Sprague-Dawley rats were stereotaxically injected in the hippocampal dentate gyrus (DG). Group I (n = 5) received GrB injection in the DG and clofazimine (50 µg/kg/day) intraperitoneally for ten days (three days before and seven days after the GrB injection). Group II (n = 4) received GrB (1 µg/µl) injection in the DG and vehicle by intraperitoneal injection. Group III (n = 4) received control solution both by DG injection and intraperitoneally. Brain sections were then immunostained for DCX-positive cells (red) in rat DG. DCX-positive cells were then counted and presented as average ± SEM.

### Clofazimine Protected Neurons from GrB-induced Neurotoxicity *in vivo*

Clofazimine is an FDA approved anti-mycobacterial drug and was found to inhibit the Kv1.3 channel [34]. It easily penetrates the blood-brainbarrier (BBB), reaches the brain, and is thus, a clinically useful candidate for Kv1.3 inhibition. We studied the effect of clofazimine on GrB-induced neurotoxicity *in vivo* by detecting its effect on the DCX-positive cells in rat dentate gyrus (DG). DCX is expressed almost exclusively in newly generated immature neurons [33], and is a marker for *in vivo* neurogenesis. Clofazamine was administered 3 days prior and 7 days after GrB injection. We found GrB significantly decreased the number and neurite length of DCX-positive cells compared to controls, while clofazimine completely blocked the effect (Figure 7).

### Kv1.3 Expression in Neurons of MS Patients

**Figure 8:**
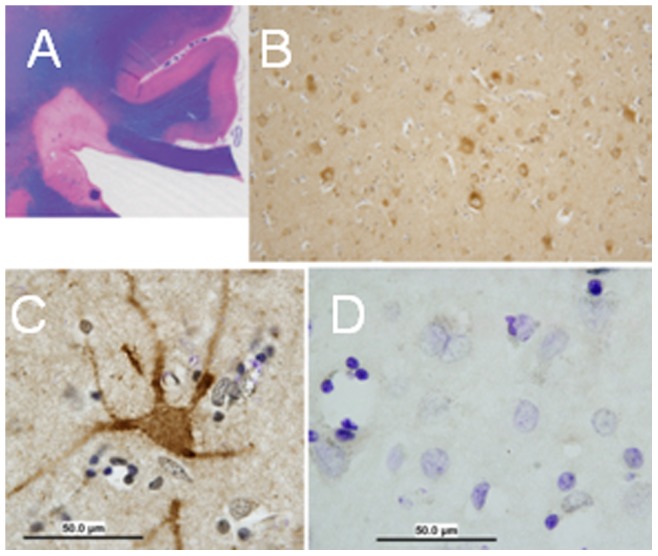
Immunostaining for Kv1.3 in autopsy brain tissue in patients with multiple sclerosis. (A) Section presents an area in the cortex showing a demyelinating lesion using hematoxylin and eosin staining. (B) Several neurons are seen within this lesion immunostaining for Kv1.3. (C) At higher magnification, cell body and neurites are immunostained for Kv1.3. (D) Staining with primary antibody only shows absence of immunostaining.

Immunostaining for Kv1.3 in brain tissues obtained at autopsy from MS patients showed an increase in neuronal immunoreactivity in focal areas of the cerebral cortex adjacent to areas of subcortical white matter demyelination (Figure 8A). In these regions, the pattern of Kv1.3 expression was most prominent in the neuronal perikarya, membrane and dendrites (Figure 8B &C). Few reactive astrocytes had mild immunostaining but no oligodendrocytes were immunostained. No staining was seen in the control tissues (Figure 8D).

## Discussion

**Figure 9:**
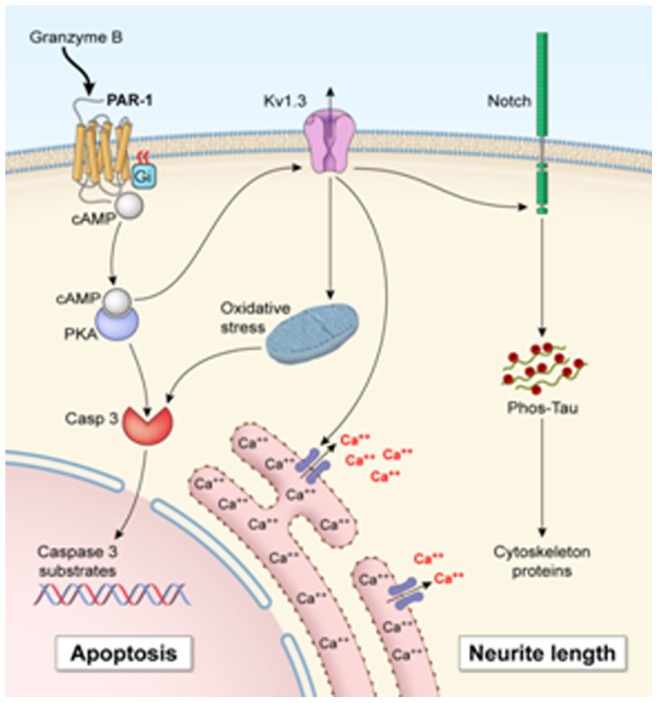
Schematic flow chart depicting possible mechanisms involved in GrB-mediated neuronal damage. Activated T cells release GrB into the supernatants. GrB induces neurotoxicity by binding and cleaving PAR-1 receptor. Activation of Gi-coupled PAR-1 then results in decreased cAMP levels, leading to Kv1.3 activation. Activation of Kv1.3 results in mitochondrial dysfunction and caspase-3 activation, which may lead to apoptosis. Kv1.3 activation can also increase Notch-1 activation, resulting in neurite damage.

We have previously shown that activated T cells-released GrB could induce toxicity in both neurons and neural progenitor cells independent of perforin [6], [20]. In the present study, we used primary cultures of human neuronal cells to investigate the mechanisms of GrB-induced neurotoxicity. We observed that GrB binds to the cell membrane receptor PAR-1 and activates the receptor by cleavage, resulting in decreased intracellular cAMP level and activation of Kv1.3 channel. This was followed by activation of the Notch-1 pathway, leading to damaged neurons (Figure 9).

As a proteinase stored in granules in mainly cytotoxic T cells and NK cells, GrB is a critical component released by activated T lymphocytes that induces cytotoxicity. It plays essential roles in inhibiting viral infections and tumors [35]. However, it is also thought to cause transplantation rejection, autoimmune diseases, and inflammation-related neurodegeneration [36]–[38]. The actual pathway of perforin-independent GrB effect has yet to be clarified and it is possible that GrB induces cytotoxicity through multiple mechanisms depending on different cell types and cell conditions. Previously, we observed that GrB caused cell death in human neurons independent of perforin and mannose-6-phosphate receptors but instead, through a PTX-sensitive pathway, suggesting stimulation of Gi coupled receptors [6]. We now demonstrate that the effect of GrB on retraction of neurites is more profound. Hence, to study the mechanisms underlying GrB-induced neurotoxicity, we focused mainly on the GrB-induced effect on neurite length as an end point since this gave us a wider detection window to measure the effects of various pharmacological interventions.

Using co-immunoprecipitation, we found that GrB binds to membrane Gi coupled receptor PAR-1 within 30 min. The almost immediate decrease of PAR-1 protein levels after GrB treatment indicates degradation of the protein instead of inhibition of protein production. Also, it is in agreement with PAR-1 activation by cleavage as it has been reported that in comparison with other GPCR proteins, activated PAR-1 receptor is not recycled but transferred to lysosome and rapidly degraded, resulting in the decreased level of the protein [39]. Inhibition of PAR-1 activation by SCH attenuated the effects of GrB on intracellular cAMP levels and neurite damage, indicating PAR-1 activation-mediated GrB effects on neurons. PAR-1 expression has been previously characterized in neurons and glial cells [14]. Inhibition of PAR-1 activation by either gene knockout or pharmaceutical inhibitors is protective in MPTP-induced dopaminergic neural terminal damage in a mouse Parkinson’s disease model [14], which is consistent with our observations. Gi coupled receptors are known to regulate adenylyl cyclase which in turn regulates cAMP levels. This may have important implications for neuronal function since as a secondary messenger, cAMP plays a critical role in synaptic plasticity and long-term memory formation [40], [41] as well as neurogenesis. That GrB binding and activation of PAR-1 receptors initiate the GrB activation pathway is a novel finding.

Another novel observation in the present study is that the Kv1.3 channel plays an important role in mediating the GrB effect. K+ plays a critical role in maintaining the cellular ion homeostasis and cell volume [42] and the enhancement of the plasma membrane permeability to K+ ions has been associated with apoptotic stimuli in a number of cell types including neurons [43]. We observed that GrB treatment caused a significant decrease in intracellular K^+^ concentration compared to the control neurons, but not in neurons pretreated with MgTX, indicating the depletion of intracellular K^+^ through the Kv1.3 channel may play a role in GrB-induced neurotoxicity. GrB treatment also increased Kv1.3 expression in a subset of neurons, in both soma and neurites. A similar pattern of Kv1.3 expression was also seen in cortical neurons from MS patients within chronic active inflammatory lesions. This effect was regulated at the level of transcription and required new protein formation. The effect of GrB was specific for this channel since inhibition of Kv1.3 but not of a variety of other K channels attenuated the effect of GrB mediated neurotoxicity. Interestingly, the increased Kv1.3 could be attenuated by treatment of PAR-1 siRNA. PAR-1regulation of Kv1.3 has not been shown before. Furthermore, blocking Kv1.3 with MgTX resulted in attenuation of GrB-induced Notch-1 activation and neurotoxicity. Importantly however, although GrB treatment increased Kv1.3 production at 24 hr as shown by immunostaining and Western blot analysis, the effect on neurotoxicity does not necessarily depend on increased Kv1.3 protein production but rather on channel activation. This effect was specific for GrB since neurotoxicity by other agents such 3-NP and 6-OH dopamine could not be blocked by Kv1.3 antagonists. Significant Notch-1 activation was observed as early as 3 hr after GrB treatment and inhibition of Kv1.3 activity by MgTX largely attenuated Notch-1 activation which otherwise would result in neurotoxicity. Thus, the neuroprotective effect of MgTX was observed before Kv1.3 protein levels increased. Our observation that cAMP may regulate Kv1.3 activation is also supported by previous studies. For example, increased cAMP can result in TrkB activation either directly or through release of brain-derived neurotrophic factor [44]-[46]. Additionally, activation of TrkB can cause phosphorylation of multiple tyrosine residues in the N and C terminals of the Kv1.3 channel protein which suppresses Kv1.3 activation [47]. Thus, a decrease in cAMP level by GrB treatment could possibly result in diminished phosphorylation of Kv1.3 resulting in its activation. Kv1.3 also plays a role in axonal guidance in the mouse olfactory system in a state-dependent manner [48], [49]. Kv1.3 is not only expressed on the cell surface but also on the mitochondrial membrane and the Golgi apparatus [50], [51]. Since it is also known that pre-Notch-1 is assembled and modified in Golgi as well [52], this could be a potential site for interactions between the two molecules. However, while fluconazole was protective against both GrB and 3NP-induced neuronal apoptosis and both 3NP and fluconazole showed no significant effect on Kv1.3 expression, it is likely that the GrB-induced Kv1.3 expression occurred before mitochondrial inhibition. Furthermore, Kv1.3 can actually modulate receptor-linked tyrosine kinase expression and activity such as TrkB kinase and related insulin receptor kinase [47]. Thus, Kv1.3 has a potential to modify intracellular events through its activation in cell membrane, mitochondria and Golgi. It is also possible that Kv1.3 may act as a secondary messenger in controlling neurite functions, in this case, through regulating Notch-1 activation.

Notch-1 activation is important in cell fate selection throughout development. Notch-1 is a membrane-bound receptor and its activation requires the proteolytic release of the NICD, which interacts preferentially with the CSL family of DNA-binding proteins, resulting in transcriptional changes in the nucleus [53]. Activation of Notch-1 is responsible for neuronal process shrinkage and dendritic atrophy in prion disease [11] and can be rescued by inhibition of North-1 activation [12]. Increased Notch-1 expression has also been observed in the hippocampus from patients with Alzheimer’s disease and fronto-temporal lobe dementia or Pick’s disease, where abnormal tau aggregates are present [54]. Interestingly, abundant Notch expression was also noted in inflammatory/demyelinating lesions in MS and its animal model, experimental allergic encephalomyelitis, and is thought to play an important role in mediating disease progression and is thus a potential therapeutic target [55]. Notch-1 transcripts are down-regulated by inducers of cAMP, suggesting an interaction between cAMP level and Notch-1 activation [56]. While Notch-1 activation is necessary for maintaining neural progenitor cells, inhibition of Notch-1 has been attributed to neuronal differentiation [57]. Our observation that Notch-1 activation occurred in GrB-treated neurons with shrinkage of neuronal processes and that inhibition of Notch-1 activation attenuated the effect agrees with these previous reports. Although a previous study suggested that GrB could directly cleave Notch-1, the fact that inhibition of Kv1.3 completely attenuated GrB-induced Notch-1 activation, suggested this is unlikely to be the case in our system. Rather, an indirect pathway involving Kv1.3 activation is necessary in the GrB-activated Notch-1 pathway in neurons. Activation of Notch-1 has been associated with tau aggregation and function [54], which may result in degradation of cytoskeleton proteins including beta-III-tubulin; hence, neurite atrophy.

Since inhibition of Kv1.3 could result in both direct neuroprotection and decreased GrB release from T cells, Kv1.3 inhibitors may be the best candidate for potential therapeutic intervention for T cell associated neurodegeneration. Most specific Kv1.3 blockers, including MgTX, are not blood brain barrier permeable, and thus have limited clinical usage. Clofazimine is a known anti-mycobacterial drug and recently was found to inhibit the Kv1.3 channel [34]. It readily penetrates the blood brain barrier and reaches the brain in concentrations sufficient to completely protect against GrB-mediated neurotoxicity as observed in our *in vivo* studies.

In conclusion, we demonstrate a novel pathway through which GrB activates membrane- bound PAR-1 to cause neurotoxicity. GrB cleaves PAR-1 resulting in its activation and decreased intracellular cAMP levels which in turn activates Kv1.3 followed by Notch-1, leading to neurotoxicity (Figure 9). These observations may have important implications for T cell-mediated neuroinflammatory diseases. Using Kv1.3 inhibitors such as clofazimine may be a novel therapeutic approach for these diseases.

## Supporting Information

Figure S1 **Effect of activated T cell supernatant on axons following incubation with neuronal cell body.** Axonal fragmentation was observed in mouse cortical neurons after somal chamber was treated with human T-cell supernatant (A). No significant axonal fragmentation was observed in mouse cortical neurons after axonal chamber was treated with human T-cell supernatant (B). Axonal degeneration was not observed in control mouse cortical neurons after either chamber was treated with T-cell medium. Instead, growth was observed (C). Legend: (a) axons before treatment; (b) axons 72 hours after treatment.
(PPT)

Figure S2 **Effect of activated T cells supernatant on PAR-1 and Notch-1 activation.** Primary cultured human fetal neurons were treated with supernatants (1∶20 dilution) from CD3/CD28 activated T cells (AT) or non-activated T cells (CT) for 3 and 18 hours. PAR-1 and activated Notch-1 fragment NICD were detected by Western-blot analysis. AT treatment group showed moderately decreased PAR-1 and significantly increased NICD after 3 hours of treatment and significantly decreased PAR-1 after 18 hours, compared to CT.
(PPT)

Figure S3 **Activated T cells supernatant increased Kv1.3 expression in primary cultured human fetal neurons.** Primary cultured human fetal neurons were treated with supernatants (1∶20 dilution) from CD3/CD28 activated T cells (AT) or non-activated T cells (CT) for 18 hours. Neurotoxicity and the Kv1.3 expression were detected by immunostaining. AT treatment caused retraction of neuronal processes as evidenced by decreased β-III-tubulin staining but increased Kv1.3 expression in the damaged neurons.
(PPT)

Figure S4 **Detection of K^+^ concentration using PBFI assay.** The PBFI assay was calibrated with known extracellular K^+^ concentrations which were increased from 0 to 160 mM in 40-mM increments by substituting Na^+^ for K^+^ in non-K solution. We found that the fluorescence values detected at Ex wavelength 340 nm correlated with the extracellular K^+^ concentration.
(PPT)
