# Supplementary figures and images for: Granzyme B-Induced Neurotoxicity Is Mediated via Activation of PAR-1 Receptor and Kv1.3 Channel
Source: PLoS One. 2012 Aug 29;7(8):e43950. doi: 10.1371/journal.pone.0043950 (PMC3430617; doi:10.1371/journal.pone.0043950)

## Slide 1
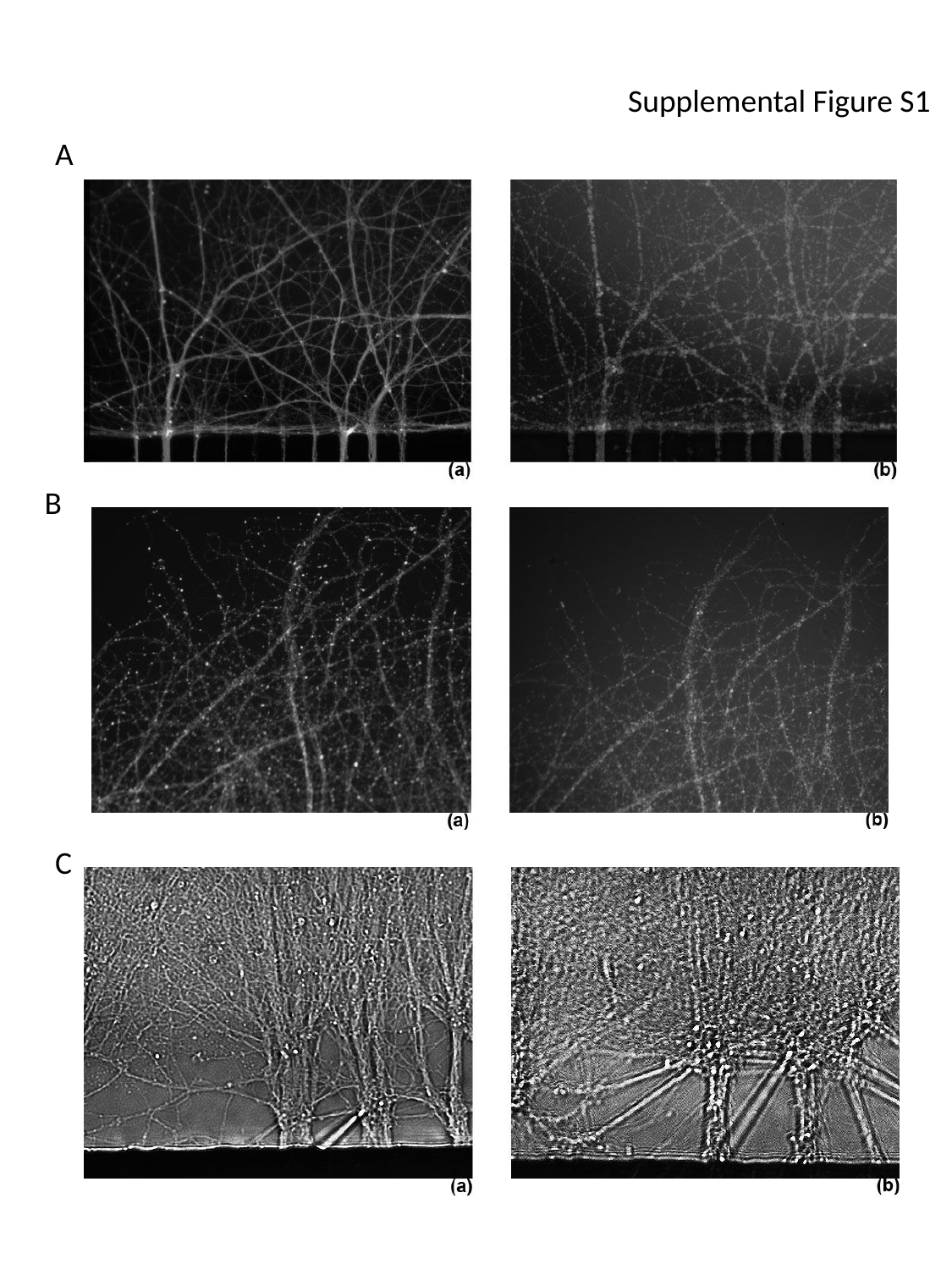

Supplemental Figure S1
A
B
C

Supplement: Figure S1 — Effect of activated T cell supernatant on axons following incubation with neuronal cell body. Axonal fragmentation was observed in mouse cortical neurons after somal chamber was treated with human T-cell supernatant (A). No significant axonal fragmentation was observed in mouse cortical neurons after axonal chamber was treated with human T-cell supernatant (B). Axonal degeneration was not observed in control mouse cortical neurons after either chamber was treated with T-cell medium. Instead, growth was observed (C). Legend: (a) axons before treatment; (b) axons 72 hours after treatment. (PPT) [file pone.0043950.s001.ppt]

## Slide 1
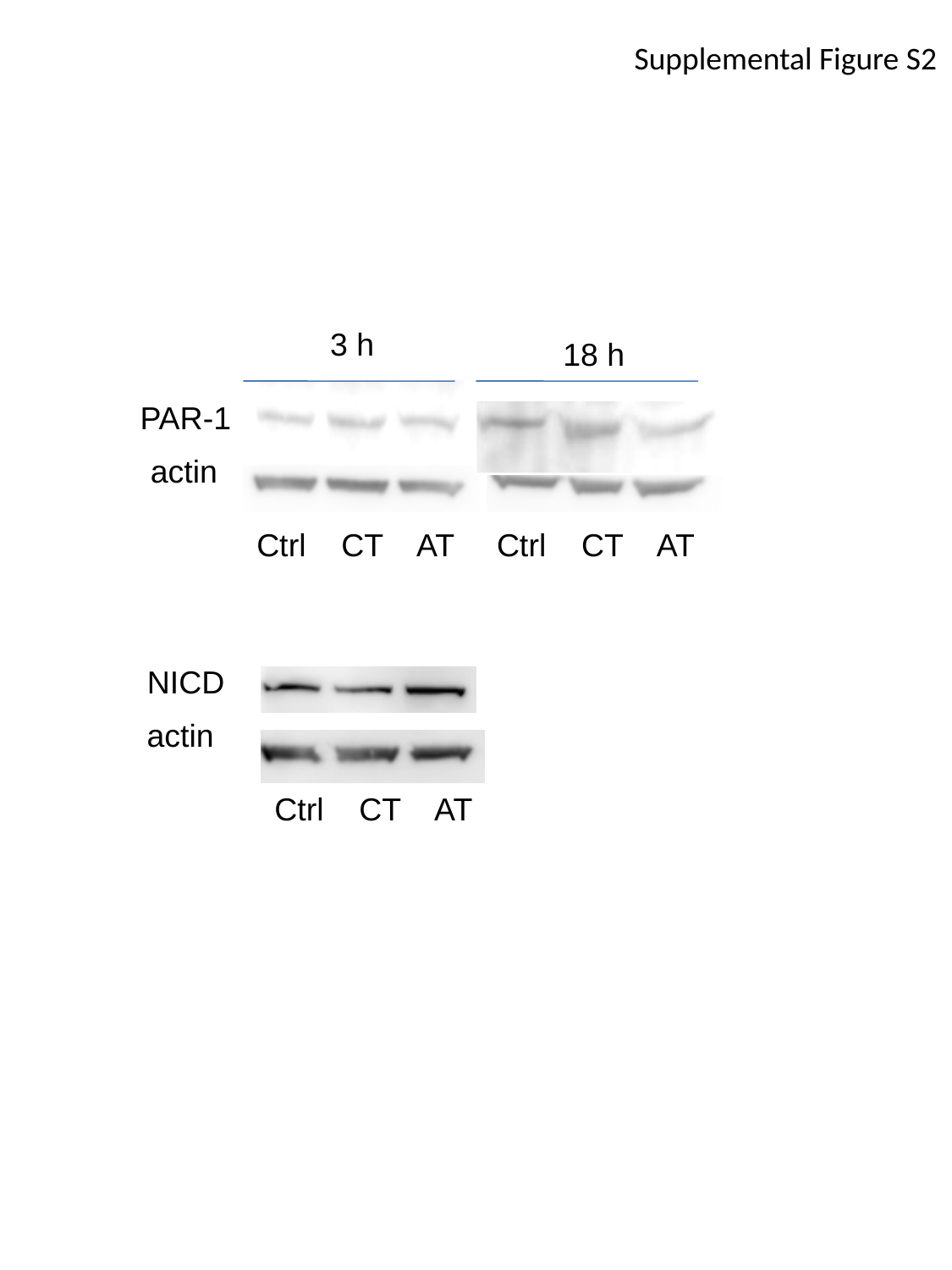

Supplemental Figure S2
3 h
18 h
PAR-1
actin
Ctrl CT AT
 Ctrl CT AT
NICD
actin
Ctrl CT AT

Supplement: Figure S2 — Effect of activated T cells supernatant on PAR-1 and Notch-1 activation. Primary cultured human fetal neurons were treated with supernatants (1∶20 dilution) from CD3/CD28 activated T cells (AT) or non-activated T cells (CT) for 3 and 18 hours. PAR-1 and activated Notch-1 fragment NICD were detected by Western-blot analysis. AT treatment group showed moderately decreased PAR-1 and significantly increased NICD after 3 hours of treatment and significantly decreased PAR-1 after 18 hours, compared to CT. (PPT) [file pone.0043950.s002.ppt]

## Slide 1
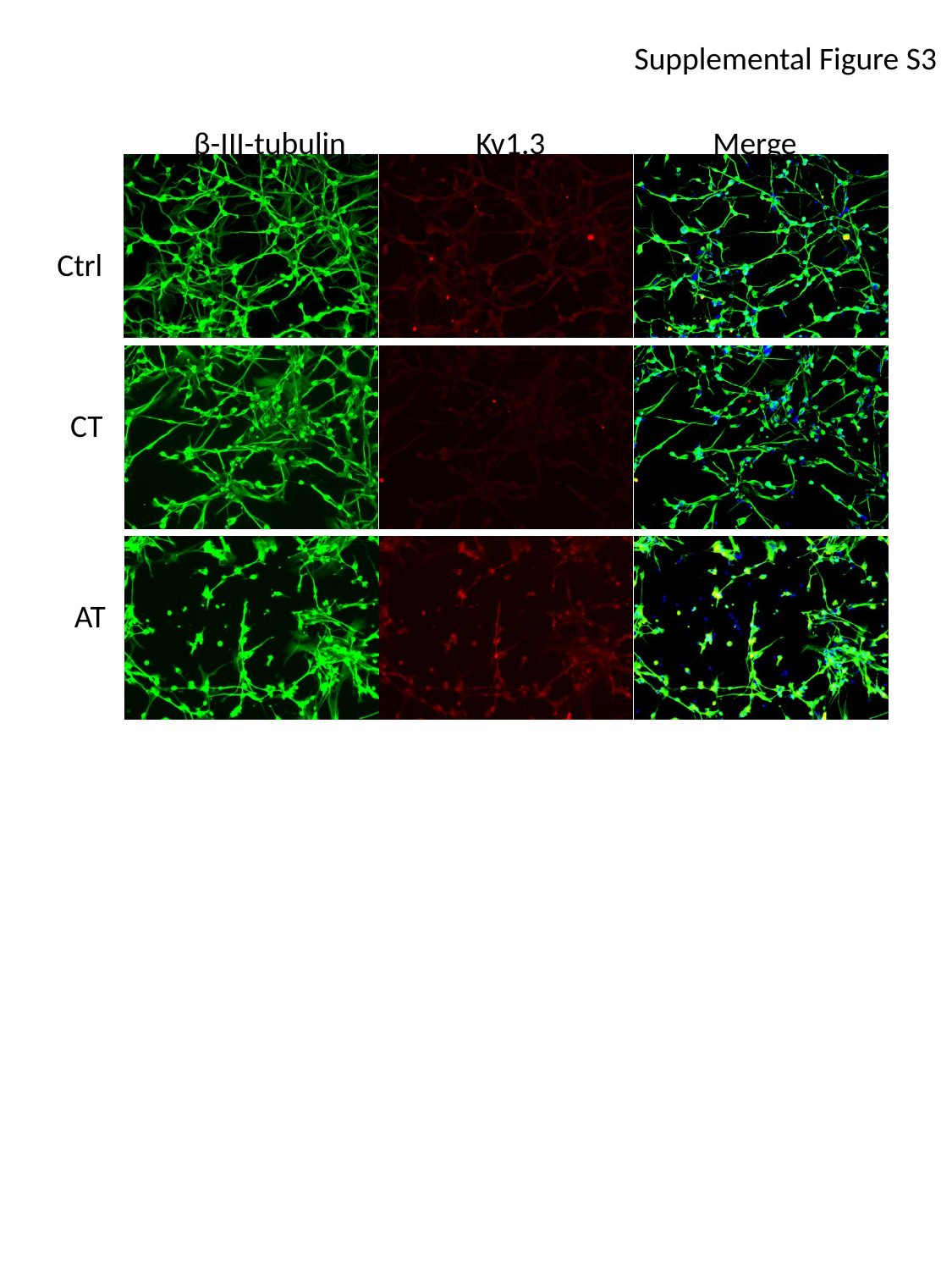

Supplemental Figure S3
β-III-tubulin
Kv1.3
Merge
Ctrl
CT
AT

Supplement: Figure S3 — Activated T cells supernatant increased Kv1.3 expression in primary cultured human fetal neurons. Primary cultured human fetal neurons were treated with supernatants (1∶20 dilution) from CD3/CD28 activated T cells (AT) or non-activated T cells (CT) for 18 hours. Neurotoxicity and the Kv1.3 expression were detected by immunostaining. AT treatment caused retraction of neuronal processes as evidenced by decreased β-III-tubulin staining but increased Kv1.3 expression in the damaged neurons. (PPT) [file pone.0043950.s003.ppt]

## Slide 1
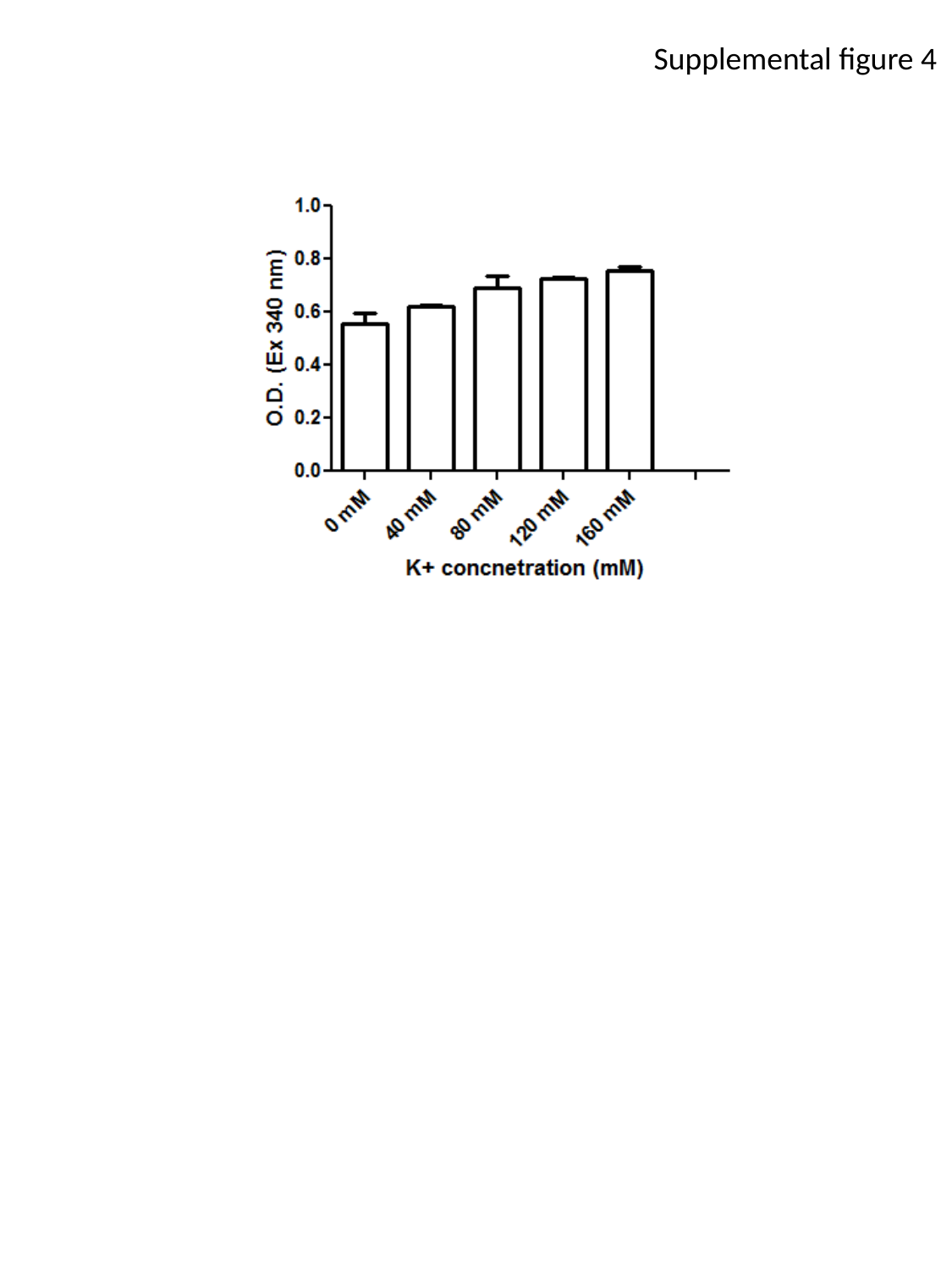

Supplemental figure 4

Supplement: Figure S4 — Detection of K+ concentration using PBFI assay. The PBFI assay was calibrated with known extracellular K+ concentrations which were increased from 0 to 160 mM in 40-mM increments by substituting Na+ for K+ in non-K solution. We found that the fluorescence values detected at Ex wavelength 340 nm correlated with the extracellular K+ concentration. (PPT) [file pone.0043950.s004.ppt]
